# Healthcare Providers' Strategies to Improve Breast Cancer Screening Among South Asian Women in Victoria

**DOI:** 10.1002/hpja.70251

**Published:** 2026-09-28

**Authors:** Farwa Rizvi, Victoria White, Patricia M. Livingston, Kylee Bellingham, Sue‐Anne McLachlan, Jennifer Philip

**Affiliations:** ^1^ Faculty of Health Deakin University Melbourne Victoria Australia; ^2^ Faculty of Health Institute for Health Transformation, Deakin University Melbourne Victoria Australia; ^3^ Department of Medicine, Palliative Nexus University of Melbourne Melbourne Victoria Australia; ^4^ Oncology and Cancer Services St Vincent's Hospital, University of Melbourne Melbourne Victoria Australia; ^5^ Palliative Medicine, University of Melbourne and St Vincent's Hospital Melbourne, Peter MacCallum Cancer Centre, the Royal Melbourne Hospital, Victorian Comprehensive Cancer Centre (VCCC Alliance) Melbourne Victoria Australia

**Keywords:** breast cancer screening, communication, cultural competency, health literacy, primary healthcare, South Asian women

## Abstract

**Background:**

Participation in breast cancer screening among culturally diverse women in Australia remains below national targets. Healthcare providers, particularly general practitioners (GPs), play a pivotal role in bridging communication and knowledge gaps and in advocacy, especially for under‐screened South Asian (Pakistani and Indian) women.

**Objective:**

To explore communication and health education strategies used by healthcare providers to encourage breast cancer screening among culturally diverse South Asian women living in Victoria.

**Methods:**

A qualitative study involving semi‐structured interviews with 15 GPs and non‐primary healthcare providers in Victoria, Australia. Participants were recruited using purposive and snowball sampling methods, drawing from primary care, specialist networks and peer‐to‐peer referrals. Reflexive thematic analysis was applied to identify communication strategies and system‐level insights.

**Results:**

Three major themes were identified: (1) encouraging health engagement; (2) communication strategies; (3) cultural sensitivity of healthcare providers. Trust‐building strategies were important in encouraging health engagement and reducing resistance to screening and misconceptions. Participants reported low levels of health literacy, including limited awareness of BreastScreen Victoria's free service. While interpreters aided communication, they also posed challenges in maintaining efficient consultations. Healthcare providers promoted patient‐centred, visual resources to support screening discussions. Cultural sensitivity was emphasised through the use of culturally competent tools and healthcare providers' ongoing cultural training. Existing screening informational resources were considered insufficient to address the cultural preferences of screening South Asian women.

**Conclusions:**

Strengthening breast cancer screening for culturally diverse women relies on building trust through culturally competent healthcare providers who can support their patients' engagement with screening through empowering communication, tailored tools and shared decision‐making.

**So What:**

Healthcare providers are key enablers of breast cancer screening participation among South Asian women. Investing in culturally competent communication, tailored resources and workforce training could substantially improve screening engagement among South Asian women who remain under‐screened despite access to free screening services.

## 1 Introduction

Breast cancer is the most commonly diagnosed cancer among women globally, with an estimated 2.3 million new cases reported in 2020, representing nearly one‐quarter of all female cancer diagnoses [1]. Early detection through regular breast screening significantly improves survival outcomes, yet uptake varies widely across population groups and healthcare systems [2].

Australia offers free breast screening mammography via a national screening programme to eligible women through structured, invitation‐based systems [3], where women aged 50–74 years receive invitations, but women aged 40 and over can attend for breast screening. Research has found that Australia's national breast screening programme, BreastScreen Australia, has halved deaths from breast cancer from 74 to 38 per 100 000 between 1991 and 2021 among women aged 50–74 [4]. Despite this, participation remains suboptimal, with the most recent data suggesting 52% of eligible women participated in 2024, which is slightly lower than participation rates pre‐COVID (53%–54%) [5].

Factors such as age, ethnicity, religious beliefs and geographical location significantly influence screening behaviours [6]. Participation among migrant women from culturally diverse backgrounds remains disproportionately low. In 2022–2023, participation among women aged 50–74 in Australia was 39.9% for those speaking a language other than English at home, compared with 53.9% for English speakers [5]. Lack of participation in screening programmes has meant that outcomes for breast cancer are worse for women from culturally and linguistically diverse (CALD) groups. In South Asian communities in particular, later‐stage diagnosis is more common, often necessitating more aggressive treatment and leading to poorer prognoses [7]. These disparities are influenced by cultural beliefs, limited health literacy, language barriers and low perceived cancer risk [8, 9, 10]. Additionally, a European study across 17 countries emphasised that without effective recruitment strategies and tailored approaches for under‐screened populations, further improvements in participation rates are unlikely [2]. With over 30% of Australia's population [11] identifying as culturally diverse [12], improving screening uptake in these groups is a public health priority.

Healthcare provider endorsement has been recognised as important in increasing screening participation [13], and routine general practitioner (GP) consultations provide a key setting for integrating preventive care and screening. However, there is limited understanding of how healthcare providers communicate with culturally diverse women, including South Asian women, about breast cancer screening. This study therefore sought to explore healthcare providers' perceptions of breast screening for South Asian women in Victoria, including their views of challenges, enablers and their approach to communicating about this screening test. The outcomes of this study will inform strategies to improve rates of breast screening in this South Asian population.

## 2 Materials and Methods

### 2.1 Study Design

This study was conducted as part of the Breast Cancer Education and Awareness of Mammography in Victoria (BEAMVIC) programme, which explored breast cancer screening among culturally diverse populations in Victoria, Australia [14]. Between October 2023 and December 2024, we conducted once‐only qualitative semi‐structured interviews [15] with 15 healthcare providers, either in person or via Zoom [16]. A descriptive qualitative methodology was used to explore participants' views and experiences. This approach aimed to provide a rich understanding of healthcare providers' views on engaging women from South Asian communities in discussions about breast cancer screening.

### 2.2 Data Collection

The 15 participants included GPs, as well as healthcare providers specialising in delivering breast screening or breast cancer treatment, including radiologists, radiographers, breast surgeons and oncologists. All had experience working with culturally diverse populations, including South Asian communities. Interviewing GPs and specialist healthcare providers was valuable as they play essential roles in screening and care in public and private clinics [8, 17]. Eligibility criteria included current medical registration with the Medical Board of Australia through the Australian Health Practitioner Regulation Agency (Ahpra) and a minimum of 3–5 years of clinical practice in Victoria.

Purposive and snowball sampling were used to recruit participants through primary care networks, peer‐to‐peer recommendations and social media. The study flyer included a brief invitation and researcher contact details. Interested healthcare providers emailed the researcher and received the participant information and consent form. Written consent was obtained before scheduling interviews, and verbal reconfirmation was provided at the start of each interview. Semi‐structured interviews (15–20 min) were conducted in English via the Zoom platform for 12 participants and in person for 3 GPs. All interviews were audio‐recorded with permission and continued until thematic saturation was reached. Participants were assured of confidentiality and de‐identification. A structured interview guide comprising four to five key questions was used, which explored broad domains including communication approaches to breast screening, perceived barriers and misconceptions, strategies to encourage screening participation, experiences engaging first‐time and repeat screeners, recommendations for improving awareness and suggested policy and system‐level improvements. The full interview guide is provided in Data S1.

### 2.3 Qualitative Data Analysis

Audio recordings of the interviews were transcribed verbatim and thematic analysis was undertaken. Themes were developed using an inductive coding approach, guided by Braun and Clarke's reflexive thematic analysis [15], to systematically identify key patterns or themes related to communication practices and broader healthcare system dynamics. The codes and themes emerged directly from the data during analysis (inductive approach), leading to sub‐themes and major themes, enabling both structured exploration and openness to new insights. Interview transcripts were systematically examined to identify patterns, key ideas and recurring concepts, with concise codes assigned to relevant text segments. Transcripts were colour‐coded, and similar excerpts were grouped under broader thematic categories, leading to the identification of minor and major themes. A coding tree was maintained in an Excel spreadsheet to document the structure and explore connections between themes, supporting the development of an overarching narrative and capturing the interrelated, evolving nature of the findings.

To ensure the veracity of the codes, the primary researcher (F.R.) read and re‐read the interview transcripts and completed the initial coding tree. The second researcher (K.B.) re‐read the transcripts and the coding tree (Excel sheet) to verify the codes and themes. A collaborative approach was employed in which both researchers compared and discussed their findings to resolve any discrepancies, with final codes reviewed by both researchers before being incorporated into the analysis, thereby ensuring consistency and reliability in the thematic analysis. Rigour was enhanced through verbatim transcription, independent review of coding and themes, consensus discussions, an audit trail and researcher reflexivity.

### 2.4 Positionality and Reflexivity

As a female medical doctor and postdoctoral research fellow, the principal investigator had the necessary expertise to interview the healthcare providers and handle the topic of breast cancer awareness and screening discussion. The emerging data were interpreted with an awareness of Australia's specific healthcare context, policies and cultural considerations to ensure relevance and insight [15].

## 3 Results

### 3.1 Participants' Background

Interviews were conducted with 15 primary and non‐primary healthcare providers, all of whom were female doctors based in Victoria, Australia. Most were GPs (60%), with others including radiologists, radiographers, breast surgeons and breast oncologists. The majority (60%) had 10–15 years of professional experience (Table 1).

**TABLE 1:** Sociodemographic details of 15 general practitioners and non‐primary healthcare providers for strategies to improve breast cancer screening among South Asian women in Victoria, Australia (2023–2024).

Characteristics	*n* (%)
Total number of healthcare providers	15 (100%)
GPs	9 (60%)
Specialist healthcare providers^a^	6 (40%)
Years of experience in practice	
3–9	3 (20%)
10–15	9 (60%)
> 15	3 (20%)

^a^ Specialist healthcare providers included radiologists, radiographers, a breast surgeon and a breast oncologist.

Three major themes emerged from the analysis, each consisting of several subthemes. The findings highlight the interconnected strategies for improving screening participation, centred on (1) encouraging health engagement, (2) communication strategies and (3) cultural sensitivity of healthcare providers (see Table 2).

**TABLE 2:** Major themes and subthemes of a qualitative study of 15 general practitioners and non‐primary healthcare providers for strategies to improve breast cancer screening among South Asian women in Victoria, Australia (2023–2024).

	Major themes	Subthemes	Description	Illustrative quote
1	Encouraging health engagement	Trust building Perceived reasons for resistance and misconceptions Initiating patient engagement for initial and ongoing screening Leverage of family/community	Healthcare providers emphasised building trust, addressing misconceptions, encouraging ongoing screening discussions and engaging family and community to support informed participation	‘I think we as GPs definitely have a role in raising awareness in the primary practice, and, uh, being a trusted source of information for patients’. (GP9)
				‘I said, that's not the case. It's not very painful’. (GP 5)
				‘I think also emphasising that people do not necessarily need to have symptoms to have a problem and the benefit that there is in catching these diseases early’. (GP 6)
				‘I think that we need to sensitise the family, especially the husbands, about the importance of breast screening’. (GP 8)
2	Communication strategies	Communication challenges (role of interpreters and family members) Culturally tailored information Raising awareness about free service SMS reminders	Healthcare providers described using visual and multilingual resources, interpreters, family support and reminder systems to improve communication and increase awareness of BreastScreen Victoria	‘Sometimes we bring in other family members to have those discussions. I've had patients who are more comfortable coming in with a sister, a daughter or a close friend, which helps a lot’. (GP 9)
				‘I know we try to make the information available, but, you know, if the resources aren't, in a format that's easily understood or easily accessible, then it's just not as effective’. (GP 9)
				‘And so you really try to put it out there and say that look, you were lucky you can get it all free here (under BreastScreen Victoria program)’. (GP 4)
				‘Like an e‐message sort of thing, a text message on a mobile (for screening reminders)’. (HCP 13 breast oncologist)
3	Cultural sensitivity of healthcare providers	Cultural sensitivity Ongoing cultural training Cultural diversity in healthcare leadership	Participants highlighted the importance of culturally safe care, ongoing cultural competency training and leadership that reflects the diversity of the community	‘Better integration of cultural competency into our healthcare system’. (GP9)
				‘I'll talk about cultural awareness training. And I guess that's always, well, that's never going to hurt anyone’. (HCP 15 breast surgeon)
				‘There's a huge disconnect between what we should be doing and what is being done, for cultural diversity in healthcare. And obviously resources and everything, that matters’. (HCP 15 breast surgeon)

The findings highlight the interconnected strategies for improving screening. Participants emphasised building trust, addressing misconceptions, fostering early and consistent engagement, leveraging family and community influence and using effective doctor–patient communication to sustain participation in breast screening.

The major themes and subthemes, along with illustrative quotes, are presented below. The illustrative quotes are provided by general practitioners as GPs and healthcare providers as HCPs.

### 3.2 Encouraging Health Engagement

Healthcare providers emphasised building trust and communication as essential to fostering breast screening participation among culturally diverse women. Many offered education and revisited discussions over time to promote effective shared decision‐making and support informed screening choices. Participants noted limited patient awareness of the free mammography services offered by BreastScreen Victoria and generally low levels of health literacy, reflected in difficulties navigating the healthcare system and understanding screening options.

Although health literacy was not explicitly defined, participants demonstrated an implicit recognition of its influence on screening engagement. Additionally, participants supported personalised, patient‐centred advice. Four subthemes were identified that highlight the role of trust and strategies used to encourage engagement with breast screening.

#### 3.2.1 Trust Building

Healthcare providers described using proactive education and rapport‐building to foster trust and encourage breast screening participation, with this viewed as especially important for culturally diverse women.

So, when we do have the opportunity to bring up breast cancer screening, it's about being proactive. A call from a health professional they trust, those kinds of things could improve attendance rates. (GP9)

Participants recognised that more than one discussion might be needed for breast screening, while difficulties in ensuring patients follow‐up were also recognised.

I ask my patients for a follow‐up discussion if they are not ready for their breast screening. However, sometimes convincing my patients is not, uh, easy, (it) takes time. (GP 8)

#### 3.2.2 Perceived Reasons for Resistance and Misconceptions

There was recognition that disparities and avoidance in screening were often not only due to lack of awareness but also reflected deeper psychological and cultural concerns. GPs identified disparities in screening participation arising from lower prioritisation of preventive care, limited knowledge about screening procedures, fear of stigma and perceived treatment costs.

When it comes to health, preventive health, it can sometimes be lower on the priority list. People may be focusing on things other than attending these appointments. If they're working full time and if they have children and they may have financial stresses. And can't afford to miss work. (GP 6)

They also noted that many women were unaware that BreastScreen Victoria screenings are conducted exclusively by female staff.

I try to make sure to tell my patients that there's (only) female staff available to talk about the process or help with the mammogram. It's just, sometimes, it's just a matter of making them feel a little more comfortable. (GP 9)

Healthcare providers endeavoured to overcome these barriers by providing clear information sensitive to women's concerns, normalising the process and alleviating apprehension about patients' fear of discomfort and pain related to mammography procedure as well as fear of potential diagnosis and modesty.

Topics like pain and the way patient perceives the pain is very, very different to our standard medical pain assessment tools…. (GP 3)

I think education around how curative, curable breast cancer is if found early… So I think education that it's very short‐term, minimal discomfort for a potential amazing benefit if you do have a breast cancer that's diagnosed sooner rather than later. (HCP 13 breast oncologist)

#### 3.2.3 Initiating Patient Engagement for Initial and Ongoing Screening

Healthcare providers reported using routine health checks as touchpoints for advising and reassuring women to undertake breast screening.

When it's time for them to do cervical screen, and I do the women health check‐up annually, I inquire that have you done it or are you about to do the breast screening and self‐breast examination. I always advise them to do it. (GP 7)

Low health literacy about breast cancer and the Australian healthcare system may further contribute to screening disparities, and ongoing screening discussions were deemed important.

I think knowledge is, well health literacy is very important (for ongoing screening discussions) because they don't know really why they are getting the same thing every two years. (GP5)

#### 3.2.4 Leverage of Family/Community

The influential role of family and community in shaping South Asian women's decisions about breast cancer screening was recognised and was seen as one strategy that could be utilised to encourage participation in screening. Some GPs had experience of partners attending the appointments and involving them in the discussion around screening.

Sometimes involving their partners, if they're there, does help a bit because a lot of women actually do listen and of course, you know, there's a cultural aspect again, yeah, husbands play a big role in their decision making. (GP 4)

Additionally, participants noted the power of culturally and linguistically resonant communication within the community.

Conversing in their own language, when they're discussing amongst their own group, … that has more influence than you know. (GP 5)

### 3.3 Communication Strategies

Communication was key to encouraging participation in screening, with healthcare providers noting barriers and facilitators. Various communication strategies, including use of visual aids, were used to facilitate screening discussion.

Three subthemes emerged: communication challenges, the need for culturally and linguistically tailored resources and raising awareness of BreastScreen services.

I have got some charts and things there, if I need to, I will definitely bring it up and just show it (for better communication), Google it straight away and you can get all those images and just let the patient sort of discuss that. I usually just show it to them how to feel the breast and what is the difference between you know your chest wall and your ribs. (GP 4)

If they are good images or resources… I'm all for showing images or drawing diagrams for patients for effective communication. (GP3)

#### 3.3.1 Communication Challenges (Role of Interpreters and Family Members)

Several participants noted that communicating about screening goes beyond literal language and that cultural communication strategies involve more than just translating words. Additionally, participants emphasised the inclusion of family members. Participants reported facing challenges related to arranging and working with interpreters, including the role of family members as translators.

What I do is, to try to arrange for interpreters when I can, or I'll, you know, try to find resources in their language, which is not always easy. (GP 9)

You have to ensure that you've got adequately trained interpreters, which can often be a bit of an issue. And even with an interpreter, it totally changes the dynamic that you have with a patient, because it is often very hard to get the patient to talk directly to you. They talk to the interpreter. (HCP 15 breast surgeon)

#### 3.3.2 Role of Culturally and Linguistically Targeted Resource (Delivery of Information and Language)

The majority of GPs highlighted the critical need for breast screening information that is culturally appropriate and easy to understand.

Is it (information) being delivered in a culturally sensitive and sort of linguistically appropriate way as a lot of recent migrants maybe may not, or even people who have been here for decades may not sort of have the necessary English language skills. (GP 6)

Many participants highlighted the potential for community‐based cultural events to address barriers to breast screening.

I know that BreastScreen Victoria does excellent work, providing information in local languages, but in addition, the government could arrange for community forums for providing information about screening, but, in local languages, such as Urdu and Hindi. So, using different community venues would be beneficial. (GP 8)

#### 3.3.3 SMS Reminders

Some participants advocated for greater use of newer communication technologies to assist with promoting and reminding women about screening. Participants considered that government‐led initiatives involving automated text messages sent in the preferred language could be an effective strategy for boosting awareness and participation in breast screening, especially for women aged 50 and above.

The government, if they could just invest in, you know, automated text messages for women over 50, in their language and more frequent outreach, um, that would really help. (GP 9)

#### 3.3.4 Raising Awareness About Free Screening Service

Some GPs reported proactively discussing breast screening with their patients, highlighting the availability and benefits of free screening through BreastScreen Victoria. They emphasised the importance of early detection, encouraged self‐examination and directed patients to credible resources such as the BreastScreen Victoria website.

I usually talk to them about the breast screening, that it's free and let them know to check the breast screen Victoria's website for more information. (GP 8)

Women who don't have a Medicare card can certainly still access the program. (HCP 10 radiologist)

Several providers indicated they emphasised to women aged over 50 that they would be invited to participate every 2 years and highlighted the value of reinforcing that screening is free and does not require a doctor's referral, and using this as a motivator to overcome hesitation and promote a prevention‐focused mindset.

I told her that once she turns 50, she will be contacted (by BreastScreen Victoria) for mammography after every 2 years. (GP 2)

### 3.4 Cultural Sensitivity of Healthcare Providers

All participants emphasised the need for cultural sensitivity in addressing barriers to breast screening, such as reluctance to discuss breast health, fear of pain and apprehension about undressing, with these barriers seen particularly among women from conservative backgrounds.

Three areas were highlighted in subthemes: ensuring women feel culturally safe and using culturally competent tools; providing cultural training to healthcare providers and greater cultural diversity in health system leaders. Existing informational resources were considered insufficient to address the cultural safety needs of South Asian women.

#### 3.4.1 Ensuring Women Feel Culturally Safe

GPs highlighted the importance of creating a non‐judgemental and understanding environment where women feel comfortable and culturally respected during breast screening discussions.

It's about making sure that, you know, women feel comfortable, and they don't feel like they're being judged or, you know, misunderstood. (GP 9)

They noted ensuring comfort, confidentiality and awareness of female staff availability for mammograms was crucial.

I try to make sure to tell my patients that there's female staff available to talk about the process or help with the mammogram. It's just, sometimes, it's just a matter of making them feel a little more comfortable. (GP 9)

#### 3.4.2 Ongoing Cultural Training for Healthcare Providers

Several participants discussed the need for cultural training, highlighting that training should focus on culturally sensitive outreach, appropriate messaging and ensuring that healthcare providers understand and respect diverse cultural backgrounds.

Ensuring our health services are equipped to handle the unique needs of diverse communities. (GP9)

The risk assessment tools (for breast cancer risk assessment) within Australia are all very Anglo heavy validation. (HCP 15 breast surgeon)

#### 3.4.3 Cultural Diversity in Healthcare Leadership

The need for greater cultural competency in healthcare leadership and systems was emphasised by several interviewees.

I truly believe also that health leadership needs to be more diverse and reflect what we have at a population level for this to really work. (HCP 11 radiologist)

## 4 Discussion

The findings highlight healthcare providers' roles in fostering trust, delivering culturally sensitive information and employing tailored strategies to improve breast screening uptake. All participating healthcare providers were female to ensure relevance, as gender concordance can influence communication, trust and cultural sensitivity in breast health discussions, particularly among culturally diverse women where modesty and gender norms may affect comfort and engagement [8, 18, 19]. Prior research indicates that female patients may feel more comfortable discussing breast health with female healthcare providers [18], especially in communities where modesty or gender norms are prevalent.

Participants noted the importance of building trust between patients and doctors in encouraging health engagement, with perceptions that patients from culturally diverse backgrounds are more likely to trust their GP's recommendations. This aligns with literature emphasising trust and shared decision‐making in supporting informed breast screening choices [13, 20]. Our study highlighted the need for culturally sensitive communication that could provide reassurance about the mammography procedure, address fears relating to pain or discomfort and clarify that screening is conducted exclusively by female radiologists and radiographers. Such approaches were perceived to counter misinformation and enhance screening uptake among migrant women. Other studies have shown that shared decision‐making benefits from translated decision aids and co‐designed materials [21, 22] and from deliberation that respects ‘what matters most’ to patients [20, 23]. This aligns with our study's finding regarding the importance that health professionals place on culturally relevant resources to aid discussions about breast screening. GPs play a pivotal role in overcoming communication and awareness barriers. A recommendation from a trusted GP remains one of the strongest predictors of screening participation [24]. Yet, time pressures, competing clinical demands and lack of culturally appropriate resources often limit how effectively GPs can initiate conversations about screening. This highlights the importance of designing interventions, including simple informational resources that support both women and providers, addressing the broader socio‐ecological and organisational contexts in which these decisions are made.

This study highlighted some tensions around the use of interpreters. While there was recognition of the importance of professional interpreters to help ensure accurate understanding, challenges were also noted, including availability and changing the dynamic of the consultation. Use of family interpreters also provided challenges, including the risk of inaccurate or incomplete communication of information to patients. Other work from Australia has also noted the benefits and challenges of using interpreters in GPs' consultations, particularly regarding ensuring consultations remained patient‐centred, altered dynamics in consultations and trust in information conveyed. While professional interpreters supported accurate communication, issues such as limited availability and altered consultation dynamics were noted. Family interpreters also risked miscommunication. Consistent with other work in Australia [25], strategies to maintain patient‐centred consultations are needed. Patient empowerment was emphasised through clear information about BreastScreen services, including female staff, free access and reassurance about the mammography process.

Provision of culturally tailored materials in languages relevant for South Asian women was found to be important for overcoming language barriers and encouraging screening uptake. Healthcare providers in our study emphasised the need for in‐language brochures on breast cancer and screening that could be used during consultations and taken home by women for later reference. Evidence from previous reviews indicates that educational interventions are more effective in increasing screening participation when they include culturally tailored components, utilise multiple and multilevel strategies, provide language‐appropriate materials and apply personal, cognitive or model‐based approaches [26, 27, 28].

Our study highlighted the importance healthcare providers place on access to culturally competent tools that can be used to promote awareness of BreastScreen and inform women of the benefits of screening. Our participants suggested a need for linguistically targeted information materials, greater awareness of gender‐matching of staff at screening facilities, use of community events to promote screening and employing tools within the clinic that are culturally acceptable. Such strategies have been proven effective. These findings are consistent with community‐led approaches that promote culturally safe breast screening. For example, in 2018, BreastScreen Victoria, the Victorian Aboriginal Health Service (VAHS) and the Victorian Aboriginal Community Controlled Health Organisation (VACCHO) partnered to develop the Beautiful Shawl Program [29], which provides Aboriginal and Torres Strait Islander women with culturally safe and empowering breast screening experiences through a culturally responsive alternative to traditional screening pathways. Evidence indicates that culturally and linguistically tailored, co‐designed educational interventions that reframe messages to reduce stigma and raise awareness are most effective for improving breast screening uptake, particularly when integrated across individual, provider and system levels [27].

Healthcare providers in our study were aware of the need to enhance their cultural competency through ongoing, structured training and continuing professional development. Effective approaches include experiential learning through patient interactions, community engagement and reflective practice that enhances awareness of implicit biases. Participants recognised the need for designated champions within health systems to promote cultural competence and awareness among health professionals. Consistent with European recommendations, there was also a broad call for enhanced communication training through both early professional education and ongoing vocational development [30]. As reflected in recent literature [31], participants in our study noted the importance of leadership diversity in healthcare suggesting that healthcare systems should better reflect the cultural makeup of the communities they serve to ensure more inclusive and effective care.

### 4.1 Implications for Policy, Practice and Research

#### 4.1.1 Policy Implications

Policies should support the development of culturally appropriate multilingual breast screening resources, community‐based awareness initiatives, interpreter services and cultural competency training among healthcare providers to improve breast screening participation among South Asian women.

#### 4.1.2 Practice Implications

Healthcare providers should be supported with culturally appropriate educational resources and communication tools to facilitate cancer screening discussions, strengthen trust and support shared decision‐making during routine consultations.

#### 4.1.3 Research Implications

Future research should focus on the co‐design and evaluation of culturally tailored breast cancer screening resources and implementation strategies for South Asian communities and assess their impact on patient engagement and screening participation in primary care.

## 5 Limitations

This qualitative study involved a purposive sample of 15 healthcare providers in Victoria, Australia, which may limit the transferability of findings to other contexts or health systems. All participants were women, so the experiences of male doctors, particularly GPs, were not captured. Although data saturation was achieved, the perspectives captured may not reflect the full diversity of healthcare providers' experiences. As with all qualitative research, the findings are context‐specific; however, rigorous analytical procedures, including independent coding by two researchers and consensus checking, enhanced the credibility of the results. A key strength of this study lies in the richness of insights generated through in‐depth interviews, providing contextually grounded insights into strategies for improving culturally responsive breast cancer screening in primary care.

## 6 Conclusion

This study emphasises the critical role of culturally tailored communication embedded within doctor–patient relationships and trust‐building in enhancing breast cancer screening understanding and uptake among South Asian (Pakistani and Indian) women. Future work is needed to understand the facilitators and barriers to screening in women from South Asian communities and their experiences and suggestions regarding preferences for breast screening information. Future efforts should focus on improving access to culturally appropriate resources, increasing cultural competency of GPs and healthcare leadership and fostering a collaborative approach between communities and health practitioners to increase awareness of breast cancer prevention and early detection.

## Funding

This work was supported by the Shepherd Foundation (SFID: 2023‐2024), Victoria, Australia.

## Ethics Statement

Ethics approval was received from the Human Research Ethics Committee at St Vincent's Hospital Melbourne for the BEAMVIC study on 17 October 2023. Project ID: 2023/PID00215; ERM ID Number: 100205; St Vincent's Health Service: 146/23.

## Consent

Individual participants' consent was obtained as written consent prior to each interview and recorded as verbal consent as part of the interview recording.

## Conflicts of Interest

The authors declare no conflicts of interest.

## Supporting information


**Data S1:** Supplementary File 1.

## Data Availability

The data that support the findings of this study are available from the corresponding author upon reasonable request.
