# Supplementary material for: Healthcare Providers' Strategies to Improve Breast Cancer Screening Among South Asian Women in Victoria
Source: Health Promot J Austr. 2026 Sep 28;37(4):e70251. doi: 10.1002/hpja.70251 (PMC13619453; doi:10.1002/hpja.70251)
Supplement: Supplementary file 1 [file HPJA-37-0-s001.docx]

Major themes, subthemes and participants’ quotes.

|  | **Major Themes** | **Subthemes** | **Participants’ quotes** |
| --- | --- | --- | --- |
| 1 | **Encouraging health engagement** | - Trust building      - Perceived reasons for resistance and misconceptions - Initiating patient engagement for initial and ongoing screening - Leverage of family/community - Communication approaches | **Trust building and reducing anxiety**  *"I always take this opportunity to tell them, are you doing your breast check? Are you comfortable?" (GP1)*  *“I think just talking to them in a relaxed, sort of, ah, setting helps to ease their anxiety a little bit. A call from a health professional they trust, those kinds of things could improve attendance rates” (GP 9)*  *“I ask my patients for a follow-up discussion if they are not ready for their breast screening. However, sometimes convincing my patients is not, uh, easy, (it) takes time” (GP 8)*  **Perceived reasons for resistance and misconceptions**  *“When it comes to health, preventive health, it can sometimes be lower on the priority list. People may be focusing on things other than attending these appointments. If they're working full time and if they have children and they may have financial stresses. And can't afford to miss work” (GP 6)*  *“Although they would’ve been living in Australia for that long, I don't think that they (are) really ready. You know it's always, not only in this culture, but the thought of getting diagnosed with something, it's a scary thing” (GP 7)*  *“I think the mammogram is where it's a little bit more off-putting because the breasts are in compression and some women are more sensitive to pain than others. (HCP 10 radiologist)*  *“I suspect that… across not even just CALD (culturally and linguistically diverse), but all groups that you're about fear of getting a diagnosis and the implications for that. So, I think education around how curative, curable breast cancer is if found early… So I think education that it's very short-term, minimal discomfort for a potential amazing benefit if you do have a breast cancer that's diagnosed sooner rather than later”. (HCP 13 breast oncologist)*  **Initiating patient engagement for initial and ongoing screening**  *“When it's time for them to do cervical screen, and I do the women health check-up annually, I inquire that have you done it or are you about to do the breast screening and self-breast examination. I always advise them to do it” (GP 7)*  *“For some people, they may have had bad experiences with health systems and the thought of having to engage with a health system if something is found could be quite confronting.” (HCP 13 breast oncologist).*  **Leverage of family/community**  *“Sometimes involving their partners, if they're there, does help a bit because a lot of women actually do listen and of course, you know, there's a cultural aspect again, yeah, husbands play a big role in in their decision making.” (GP 4)*  *“Conversing in their own language, when they're discussing amongst their own group, maybe that that has more influence than, you know, us, you know, reaching from the platforms of, you know, the breast screen logo, you know, under that banner.” (GP 5)*  **Communication approaches**  *“But if I have got some charts and things there if I need to I will definitely bring it up (breast screening) and just show it, Google it straight away and you can get all those images and just let the patient sort of discuss that” (GP 4)*  *“I would say to the husband that look, it's important and even just put their prostate check into the conversation and say look you should get it done, just as your partner needs the breast check. And so sometimes it does work just to sort of trying to tackle it that way” (GP 4)*  *“I think also emphasising that people do not necessarily need to have symptoms to have a problem and the benefit that there is in catching these diseases early” (GP 6)*  *“I guess some explanations around pathways that look if something is found, what happens next and reassurance about support in terms of interpreters and that sort of thing” (HCP 13 breast oncologist).* |
| 2 | **Proactive preventative health communication** | - Communication challenges - SMS reminders | **Communication challenges (delivery of information, language)**  *“what I do is, is try to arrange for, ah, interpreters when I can, or I’ll, um, you know, try to find resources in their language, which is not always easy” (GP 9)*  *“How is this information being delivered? Is it being delivered in a culturally sensitive and sort of linguistically appropriate way as a lot of recent migrants maybe may not, or even people who have been here for decades may not sort of have the necessary English language skills” (GP 6)*  *“I know that BreastScreen Victoria does excellent work, um, providing information in local languages, but in addition, um, the government could arrange for community forums for providing information about screening, but, um, in local languages, such as Urdu and Hindi. So using different community venues would be beneficial” (GP 8)*  **SMS reminders**  “The government, if they could just invest in, you know, automated text messages for women over 50, um, in their language and more frequent outreach, um, that would really help” (GP 9)  “I would suggest that more and more campaigns at the GP level should be done that as soon as a woman turned 40, they should get more SMS kind of messages that have you ever thought of breast screen?” (GP 7)  “Like an e-message sort of thing, a text message on a mobile (for screening reminder)” (HCP 13 breast oncologist) |
| 3 | **Patient empowerment** | - Raising awareness about free service for screening - Culturally tailored information - Cultural events and patient equity | **Raising awareness about free service for screening**  *“I usually talk to them about the breast screening, its, um, free and let them know about monthly self-examination. I have at times asked a few women to check the breast screen Victoria’s website for more information” (GP 8)*  *“And so you really try to put it out there and say that look, you were lucky you can get it all free here through Medicare” (GP 4)*  **Culturally tailored information**  *“I know we try to make the information available, but, you know, if the resources aren't, in a format that's easily understood or easily accessible, then it’s just not as effective” (GP 9)*  *“And I actually think that the challenge for us as health professionals is that we underserve these populations, because the messages are not written for these types of (cultural) cohorts” (HCP 11 radiologist)*  **Leveraging cultural events for patient equity**  *“If you're focusing on the indoor park, I still believe that will be very good because women like to talk about these things with other women and sometimes, they feel a little bit reluctant talking to their GP and sometimes” (GP 4)*  *“Having people delivering the message who actually understand the nuance around it, I think would sort of send a stronger message” (GP 6)*  *“the government could arrange for community forums for providing information about screening, but, um, in local languages, such as Urdu and Hindi. So using different community venues would be beneficial” (GP 8)* |
| 4 | **Cultural sensitivity of healthcare providers** | - Cultural sensitisation - Ongoing cultural training - Cultural diversity in healthcare leadership | **Cultural sensitisation**  *“They [women] are reluctant to talk about certain aspects of their health, especially, you know, anything that’s related to reproductive organs or breasts, um, that’s, that’s a barrier” (GP 9)*  *“There's always been a little bit of apprehension, especially I think around the idea of sort of getting undressed or not really knowing exactly what it entails…” (GP 6)*  *“It’s about making sure that, you know, women feel comfortable, and they don’t feel like they’re being judged or, you know, misunderstood. (GP 9)*  *“I try to make sure to tell my patients that there’s female staff available to talk about the process or help with the mammogram. It’s just, sometimes, it’s just a matter of making them feel a little more comfortable” (GP 9)*  *“Topics like pain, um, the awareness of pain and the way the patient perceives the pain is very, very different to our standard medical pain assessment tools...I do feel like it because sometimes they either they are too quiet or they are too loud like two Extremes...concerns were not really taken as seriously" (GP 3)*  *“So in many of these cultures, women even in their 50s, they do not even go without their husband for a GP consult. So there's a huge cultural and religious aspect in that, and then the lower autonomy in these women” (GP 7)*  *“there's a cultural aspect again, yeah, husbands play a big role in in their decision making. So I would say to the husband that look, it's important and you know even just put their prostate check into the conversation and say look you should get it done, just as your partner needs the breast check. And so sometime it does work just to sort of trying to tackle it that way” (GP 4)*  *“Most important are the cultural factors, with some women, as they are, um, hesitant to expose their breasts for screening. Also, I think that we need to sensitise the family, especially the husbands about the importance of breast screening” (GP 8)*  *“Make it a non-judgmental environment you know sort of in their cultural background or something and get people involved in sort of conversations I think that will help to break these barriers” (GP 4)*  **Ongoing cultural training**  *“I'll talk about cultural awareness training. And I guess that's always, well, that's never going to hurt anyone” (HCP 15 breast surgeon)*  *“Better integration of cultural competency into our healthcare system. So, um, whether that’s in, training our staff or ensuring, our, our health services are equipped to handle the unique needs of diverse communities, that’s something I think is really lacking” (GP 9)*  *“I don't know if BreastScreen Victoria already has a program or something. So, you know, if it already exists, that's great. There needs to be more education when it comes to health practitioners as well” (GP 3)*  *“I would think like culturally sensitive outreach programs Is probably sort of the umbrella term for what I'm thinking is needed, and that would involve sort of language appropriate, culturally appropriate sort, messaging as well as culturally appropriate healthcare personnel delivering that care. in the way that there are sort of indigenous liaison officers” (GP 6)*  *“If all our healthcare providers had better training on cultural sensitivity, that would go a long way in improving breast screening uptake in these communities” (GP 9)*  **Cultural diversity in healthcare leadership**  *“Diversity is unifying, because each and every one of us brings something different to the table. I truly believe also that health leadership needs to be more diverse and reflect what we have at a population level for this to really work” (HCP 11 radiologist)*  *“The risk assessment tools within Australia are all very Anglo heavy validation. And in fact, even the genome wide association studies are still overly Anglo'd, which brings into question the whole, you know, even about genetic testing of people from CALD (culturally and linguistically diverse) backgrounds. There's a huge disconnect between what we should be doing and what is being done. And obviously resource, custody of resource and everything, that matters” (HCP 15 breast surgeon)* |
| 5 | Focus on family history and tailored advice | - Patient awareness of risk - Focus on family history and tailored advice | **Patient awareness of risk**  *“It would be a good idea that at 40 years of age, women get the message or letter, because breast cancer cases are happening in women in 40s or younger. So, whenever somebody turned 40 like an Australian citizen (and resident), they should send something like a small book (for self-check assessment tool) or a flyer for breast screen, make it something like as you send the reminder for cervical cancer screen every five years” (GP 7)*  *“A proper counselling (is needed) that would help them (patients) to understand the risks and importance." (GP 1)*  *"I always talk to them about breast screening even in 40 years and above since they can go and voluntarily have a screen in particularly asking if they have any, you know, risk factors or family members with positive history, if you know in terms of risk stratification." (GP 3)*  *“I usually recommend for a baseline screening to a few patients if they’re high risk in their 40s and in their 50s” (GP 8)*  **Focus on family history and tailored advice**  *“I always get a history of family history of, you know, breast cancer or breast lumps or personal history of breast lumps. So that actually helps me to formulate a plan for that woman in her 40s till she is in for the BreastScreen Victoria in her 50s” (GP 4)*  *“After inquiring their family history, if there's a risk factor or anything, I suggest screening to them” (GP 7)*  *“I think their history, the family history, yeah, any female with a family history of breast cancer. If there is if first degree relative history of breast cancer or the history of BRCA gene testing. I think it's not just the ethnicity it would also be the history” (GP 5)*  *“Knowledge is very important because they don't know really why are they getting the same thing every two years. Most of them don't even know what that is because they know that in their (host) country 20 or 25 years ago, where the biggest killer, and I think they have no concept of screening for breast cancer” (GP 5)*  *“When a woman is diagnosed with breast cancer, particularly at a younger age or with a strong family history, we always recommend genetic counselling and testing for BRCA mutations or other hereditary cancer syndromes. If the test is positive, we discuss risk reduction strategies, not only for the patient but also for their family members” (HCP 14 breast surgeon)* |
